# Green Analytical Method for Simultaneous Determination of Glucosamine and Calcium in Dietary Supplements by Capillary Electrophoresis with Capacitively Coupled Contactless Conductivity Detection

**DOI:** 10.1155/2023/2765508

**Published:** 2023-01-31

**Authors:** Yen Nhi Do, Thi Lan Phuong Kieu, Thi Huyen My Dang, Quang Huy Nguyen, Thu Hien Dang, Cao Son Tran, Anh Phuong Vu, Thi Trang Do, Thi Ngan Nguyen, Son Luong Dinh, Thi Minh Thu Nguyen, Thi Ngoc Mai Pham, Anh Quoc Hoang, Bach Pham, Thi Anh Huong Nguyen

**Affiliations:** ^1^Faculty of Chemistry, University of Science, Vietnam National University, 19 Le Thanh Tong, Hanoi 10000, Vietnam; ^2^National Institute for Food Control (NIFC), 65 Pham Than Duat, Hanoi 10000, Vietnam; ^3^Faculty of Pharmacy, University of Medicine and Pharmacy, Thai Nguyen University, 284 Luong Ngoc Quyen, Thai Nguyen 24000, Vietnam; ^4^Poison Control Center, Bach Mai Hospital, 78 Giai Phong, Hanoi 10000, Vietnam

## Abstract

The need for analytical methods that are fast, affordable, and ecologically friendly is expanding. Because of its low solvent consumption, minimal waste production, and speedy analysis, capillary electrophoresis is considered a “green” choice among analytical separation methods. With these “green” features, we have utilized the capillary electrophoresis method with capacitively coupled contactless conductivity detection (CE-C^4^D) to simultaneously determine glucosamine and Ca^2+^ in dietary supplements. The CE analysis was performed in fused silica capillaries (50 *μ*m inner diameter, 40 cm total length, 30 cm effective length), and the analytical time was around 5 min. After optimization, the CE conditions for selective determination of glucosamine and Ca^2+^ were obtained, including a 10 mM tris (hydroxymethyl) aminomethane/acetic acid (Tris/Ace) buffer of pH 5.0 as the background electrolyte; separation voltage of 20 kV; and hydrodynamic injection (siphoning) at 25 cm height for 30 s. The method illustrated good linearity over the concentration range of 5.00 to 200 mg/L of for glucosamine (*R*^2^ = 0.9994) and 1.00 to 100 mg/L for Ca^2+^ (*R*^2^ = 0.9994). Under the optimum conditions, the detection limit of glucosamine was 1.00 mg/L, while that of Ca^2+^ was 0.05 mg/L. The validated method successfully analyzed glucosamine and Ca^2+^ in seven dietary supplement samples. The measured concentrations were generally in line with the values of label claims and with cross-checking data from reference methods (HPLC and ICP-OES).

## 1. Introduction

Glucosamine (2-amino-2-deoxy-D-glucose) is an amino monosaccharide with essential roles in the biochemical synthesis of glycosylated proteins and lipids [1]. Intensive studies have proved that the exogenous use of glucosamine can relieve osteoarthritis (OA) symptoms and restore articular functions [2–5]. It also has many antiinflammatory and antioxidative effects with minimal adverse impact on human health [6–10]. Thus, glucosamine is suggested for relieving pain and preventing or slowing the breakdown of cartilage in OA by the Osteoarthritis Research Society International (OARSI) [11]. The European League Against Rheumatism (EULAR) recommended glucosamine as a treatment for knee OA [12]. The United States Food and Drug Administration (FDA) also proposed glucosamine as a dietary supplement used to manage and treat OA [13].

Because of its benefits, glucosamine products are one of the most popular over-the-counter (OTC) dietary supplements worldwide. Besides its use as a single supplement in the form of glucosamine hydrochloride or glucosamine sulfate, it can be mixed with other nutraceuticals, vitamins, and minerals. Among them, calcium is commonly combined with glucosamine because of its crucial role in preventing osteoporosis [14]. Given that there are different combinations of glucosamine and other supplements, more than one method is required to analyze these products. For example, the most popular method for glucosamine analysis is reversed-phase high-performance liquid chromatography (RP-HPLC), coupled with various detectors like ultraviolet, electrochemical, and mass spectrometry [15–19]. However, this method is not suitable for analyzing minerals like calcium, which have commonly been determined by inductively coupled plasma mass spectrometry (ICP-MS) and inductively coupled plasma optical emission spectrometry (ICP-OES), or flame atomic absorption spectrometry (F-AAS). Due to the complexity of current methods for the quantification of glucosamine products, there is a need to develop a simple technique to simultaneously determine the amounts of glucosamine and other components in the food supplement.

Capillary electrophoresis (CE) can be considered a more economical alternative for developing simple and cheap analytical techniques. CE instruments are coupled with various detection methods such as ultraviolet, laser-induced fluorescence, mass spectrometry, voltammetry, and amperometry. CE coupled with capacitively coupled contactless conductivity detection (C^4^D) has recently been highlighted as an economical and efficient method for environmental monitoring, [20–22], food control [23–25], forensics [26], and clinical analysis [27–29]. The working principles and applications of CE-C^4^D have been reviewed by Hauser and Kubáň [30] and others [29, 31–33]. These techniques have been applied to analyze inorganic, organic ions, and biomolecules. Because of the ability to analyze the broad range of targets, we have developed a protocol to simultaneously quantify both glucosamine and calcium in dietary supplements by CE-C^4^D. A simple procedure has been reported, with cross-checking data using standard high-performance liquid chromatography with a fluorescence detector (HPLC-FLD) and ICP-OES methods being provided to prove the reliability of the CE-C^4^D results. We also applied this protocol for the quality control of seven dietary supplements available in Vietnam.

## 2. Materials and Methods

### 2.1. Materials

All chemicals used were of analytical grade and were used as received without any further purification. All solutions were prepared with deionized water of resistivity not less than 18.2 MΩ cm^−1,^ which was purified by the WaterPro RO system (Labconco Cooperation, Kansas, MO, USA).

Both glucosamine and calcium nitrate were purchased from Merck KGaA (Germany). The stock solutions of glucosamine (1000 mg/L) and Ca^2+^ (1000 mg/L) and standard solutions of calibration curves were prepared with deionized water in volumetric flasks and stored at 4°C before further use.

L-arginine (Arg), N-cyclohexyl-3-aminopropanesulfonic acid (CAPS), L-histidine (His), and tris (hydroxymethyl) aminomethane (Tris) used for background electrolyte (BGE) and sample preparation were purchased from Sigma–Aldrich (Singapore). Otherwise, acetic acid (Ace) and trichloroacetic acid (TCA) were purchased from Merck KgaA, Germany. The BGEs were prepared by adding Ace into Arg, Tris, His, and CAPS solutions, followed by pH adjustment with acetic acid using a HI 2215 pH meter (Hanna Instruments, Woonsocket, RI, USA).

Fused silica capillaries (50 *μ*m i.d., 365 *μ*m OD) were purchased from BGB Analytik AG (Böckten, Switzerland). Before use, the capillaries were conditioned by flushing with 1 M NaOH solution, deionized water, and then the BGE.

### 2.2. Sample Preparation

Dietary supplement samples, including tablets, and hard capsules, were purchased from pharmacies in Hanoi, Vietnam. The sample preparation procedure was described in previous studies [34, 35]. Briefly, each sample was prepared with the contents of 20 whole tablets or capsules. The tablets were ground into a fine powder using a ceramic mortar and pestle. The hard capsules were opened to collect powder; the samples were ground if necessary. The sample was accurately weighed (about 0.5 g) before being transferred into a 25-mL volumetric flask; 4% TCA was added to the mark. The flask was ultrasonicated for 30 min by an ultrasonic vibrator bath (BRANSON 521) and centrifuged at 3000 rpm for 15 min. After centrifugation, the aqueous phases were collected and passed through a 0.45-*μ*m syringe filter. The filtrated solution was transferred to a 25 mL volumetric flask and filled to the mark with 4% TCA. The solution could be diluted with deionized water (if necessary) before analysis by CE-C^4^D. Concentrations of glucosamine and Ca^2+^ were determined by using the standard addition method.

### 2.3. Instrument

The in-house CE-C^4^D instrument was presented in the previous studies [30, 36, 37]. Generally, the compact CE instrument was built in-house using high voltages of a maximum of 25 kV (UM25*∗*4, 12 V, Spellman, Pulborough, UK) and combined with a commercial C^4^D detector (ER815, eDAQ, Denistone East, NSW, Australia). The system was operated with a 220-VAC power supply. The silica capillary had a total length (*L*_tot_) and effective length (*L*_eff_) of 40 cm and 30 cm, respectively, and an inner diameter of 50 *μ*m.

### 2.4. Method Development and Validation

Analytical parameter optimization followed strategies in our earlier works [26, 34, 35, 38, 39]. Overall, various factors of BGEs (composition, pH, and concentration), separation voltages, and injection conditions (siphoning height and injection time) were investigated to develop an effective CE-C^4^D method for the simultaneous determination of glucosamine and Ca^2+^. A single analytical parameter was changed at a time while fixing all the others. The background noise, signal, and migration times of analytes were calculated from the CE electropherograms to identify the optimized condition (Tables S1–S6). Because background noise may change during the recording of an electropherogram, the average noise was measured near the analyte peak regions. For a reproducible and accurate measurement, the signal's peak area was used in all this study's steps. After comparing the analytes' signals, background noise, and retention time under different analytical conditions, the optimized CE-C^4^D conditions are summarized in Table S7.

Based on optimized CE-C^4^D conditions, the analytical method was validated for linearity, detection and quantification limits, repeatability, and recovery by using the analytical results of standard solutions and matrix-spike samples. In pharmaceutical analysis, guidelines from the International Council for Harmonization (ICH) and EURACHEM are commonly used to calculate the limit of detection (LOD) [40, 41]. They define the LOD as the concentration of analyte that generates a signal at least 3 times the signal noise of the baseline (*S*/*N* = 3); for LOQ, the *S*/*N* ratio equals 10. Thus, in this study, to determine the LOD of each analyte (calcium or glucosamine), a standard solution was repeatedly diluted until its signal was 3 times higher than the background noise (*S*/*N* = 3). The LOQ value of each analyte was measured by repeat dilution until the signal of the diluted solution was 10 times higher than the background noise (*S*/*N* = 10). All LOD/LOQ measurements were conducted under optimum conditions in Table S7.

Cross-check analysis was performed for both sample types (i.e., tablets and capsules). Glucosamine was analyzed with HPLC coupled with a fluorescence (FLD) detector, while ICP-OES detected Ca^2+^ at the National Institute for Food Control (NIFC), Hanoi, Vietnam, according to methods reported elsewhere [42, 43]. All measurements generally followed the AOAC Official Method 2016.14. Detailed information about the reference methods was listed in Table S8.

## 3. Results and Discussion

### 3.1. Optimization of CE-C^4^D Analytical Conditions

#### 3.1.1. Effects of Background Electrolytes

**Figure 1:**
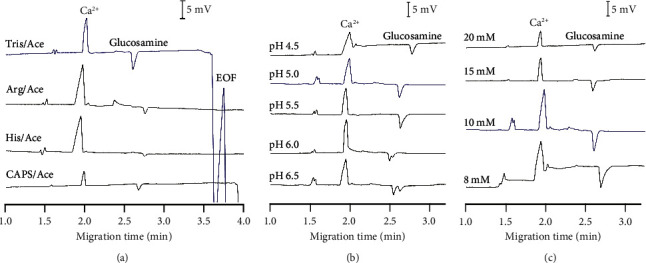
Optimization the BGE conditions for simultaneous determination of glucosamine and calcium. (a) The effect of BGE compositions. (b) The effect of pH. (c) The effect of Tris concentration. CE conditions: fused silica capillary (total length 40 cm, effective length 30 cm, ID 50 *μ*m); separation voltage +20 kV; hydrodynamic injection (siphoning at 25 cm in 30 s). The selected conditions were highlighted in blue.

The background electrolytes (BGEs) are one of the most critical factors in CE-C^4^D method development [44]. In this work, we investigated four standard BGE systems, including Arg/Ace, His/Ace, Tris/Ace, and CAPS/Ace (Figure 1(a), Table S1). The initial concentrations of the Arg, His, Tris, and CAPS solutions were 10 mM. All solutions were first adjusted to a pH of 5.0 by acetic acid. In four BGE systems, both glucosamine and Ca^2+^ were eluted before the electroosmotic flow (EOF) signal, indicating that the two analytes have positive charges under all studied conditions. Negative-going peaks of glucosamine show a conductivity reduction of the background buffer when this compound passes the detector. Although all BGE systems give a sharp peak of Ca^2+^, only the Tris/Ace solution provides a high signal of glucosamine (Figure 1(a)). Other BGE solutions cause low peak signals for glucosamine, leading to insufficient sensitivity. Thus, the BGE system containing Tris/Ace was selected because of the sufficient peak signal for Ca^2+^ and glucosamine.

After selecting the BGE composition, the pH of the solution was further investigated (Figure 1(b)). This factor significantly influences the glucosamine signal. In general, both peaks of Ca^2+^ and glucosamine were far from the EOF signals at all five pH conditions (pH 6.5–4.5). The high pH declines the migration time of both analyzes because of the rise in the EOF mobility. At pH 6.0 and higher, the peak separation of glucosamine appears, possibly due to the conversion of glucosamine forms at different pHs. While *β*-anomer is dominant for nonprotonated neutral glucosamine, the protonated glucosamine mainly exists as *α*-anomer [45–47]. The two peaks of glucosamine are more resolved in higher pH conditions. Two isomers of glucosamine also generate two peaks in HPLC analysis; thus, the sum of the area of these two peaks should be used to quantify the glucosamine [15, 48, 49]. The pH of 4.5 to 5.5 is suitable to accurately detect total glucosamine by CE-C^4^D since glucosamine generates a single peak at these conditions, leading to a more straightforward calculation. All three pH conditions gave the stable baseline and the practical time for bulk analysis (Table S2). The content of Ca^2+^ is much lower than that of glucosamine in commercial supplements; thus, a condition that shows the best Ca^2+^ signal should be chosen for the simultaneous determination of both analytes in supplement samples. Among all pH conditions, the pH of 5.0 exhibited the highest peak area of Ca^2+^ signals and a single peak of glucosamine. Accordingly, a pH of 5.0 was chosen for further studies.

Tris concentration (from 8 to 20 mM) was optimized for simultaneous determination of glucosamine and Ca^2+^ (Figure 1(c) and Table S3). Generally, the increase in Tris concentration shows a minor effect on the migration times of glucosamine and Ca^2+^. In the capillary tube, a high concentration of ions causes a change in the magnitude of the electric double layer; therefore, the sample moves unevenly, causing background noise, poor separation, and unbalanced peaks. Although high concentrations of Tris (≥10 mM) in BGE provide a stable baseline and balanced peaks, the intensity of the glucosamine peak declines at high Tris concentration, decreasing the sensitivity. Thus, compared to other conditions, the 10 mM Tris/Ace electrolyte solution was selected for further investigations because of both analytes' stable baselines and balanced peaks.

#### 3.1.2. Effects of Separation Voltages

**Figure 2:**
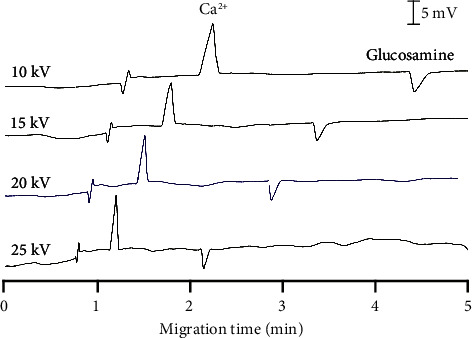
Effects of separation voltages on the CE-C^4^D analysis of glucosamine and calcium.BGE conditions: 10 mM Tris/Ace, pH 5.0, sample injection time of 30 s, sample injection height of 25 cm. The selected condition was highlighted in blue.

Various applied voltages from 10 to 25 kV were then investigated to evaluate their impacts on electropherograms of glucosamine and Ca^2+^ (Figure 2). Firstly, higher voltages resulted in shorter migration times and sharper peaks, but higher background noise was observed. Voltages of 10 or 15 kV can lead to broadening peaks of Ca^2+^ and glucosamine, which could cause false results when analyzing real sample matrices. Moreover, a high separation voltage raises the background noise (Table S4). The noise at +25 kV showed the highest level, around 2.06 mV, while the background noise was only 0.67 mV at +10 kV. On the other hand, the area of the peaks from glucosamine and Ca^2+^ decreased at a high applied voltage (Table S4). Lastly, the high voltage (higher than 20 kV) could generate audible noise, especially in high humidity conditions in a tropical country like Vietnam. Thus, it is recommended to use a low voltage in capillary electrophoresis if possible for safety. Given these points, a separation voltage of 20 kV is optimal for simultaneously determining glucosamine and Ca^2+^.

#### 3.1.3. Effects of Injection Conditions

**Figure 3:**
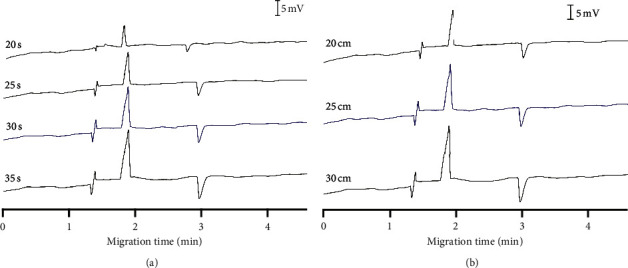
Effects of injection conditions on the CE-C^4^D analysis of glucosamine and calcium. (a) Injection time (siphoning at 25 cm). (b) Siphoning height (injection time 30 s). BGE conditions: 10 mM Tris/Ace electrolyte solution, pH of 5.0, separation voltage of +20 kV. The selected conditions were highlighted in blue.

Next, hydrodynamic injection conditions, including injection times and siphoning heights, were studied (Figure 3, Tables S5 and S6). These parameters control the sample amounts injected into the capillary, affecting the separation and detection efficiency. Peak areas are directly proportional to injection times and siphoning heights. Although longer injection times and higher injection heights can lower detection limits, too large injected sample amounts may degrade the peak shape and introduce elevated interferences. Considering these facts, we injected samples at a siphoning height of 25 cm in 30 s.

### 3.2. Method Validation

#### 3.2.1. Selectivity

**Figure 4:**
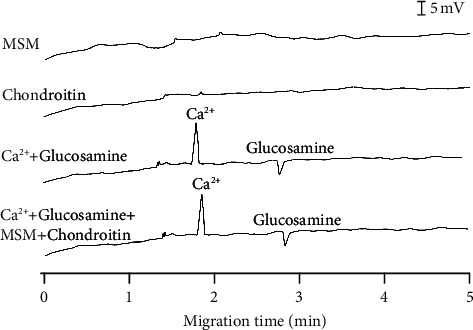
Selective determination of glucosamine and calcium in the presence of chondroitin and MSM. Concentration of calcium was 10 mg/L while concentrations of glucosamine, chondroitin, and MSM were 100 mg/L. BGE conditions: 10 mM Tris/Ace electrolyte solution, pH of 5.0, separation voltage of +20 kV, sample injection time of 30 s, sample injection height of 25 cm.

Most commercial glucosamine supplements combine chondroitin and methylsulfonylmethane (MSM) to treat painful joints. Thus, the interference of chondroitin and MSM in the signals of glucosamine and Ca^2+^ was investigated (Figure 4). There is no detectable signal for chondroitin and MSM under experimental conditions. While a positive potential was applied to detect glucosamine and Ca^2+^ (positive ions), chondroitin and MSM cannot form a positive charge under the studied condition. Chondroitin is a highly negative polysaccharide [50], and MSM, an organosulfur compound, is considered chemically inert with a pKa of 28. Thus, it is apparent that we cannot observe any signal of these two chemicals in the analyzing time (around 5 min). Moreover, the presence of chondroitin and MSM showed no interference with the peak signals of glucosamine and Ca^2+^. Thus, the proposed method is suitable for simultaneously determining glucosamine and Ca^2+^ in commercial supplements.

#### 3.2.2. Calibration Curves

**Figure 5:**
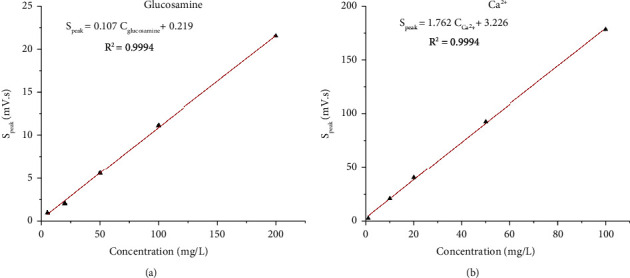
Calibration curves of glucosamine and calcium. BGE conditions: 10 mM Tris/Ace electrolyte solution, pH of 5.0, separation voltage of +20 kV, sample injection time of 30 s, sample injection height of 25 cm.

A series of mixture standard solutions ranging from 5.00 mg/L to 200 mg/L and from 1.00 mg/L to 100 mg/L of glucosamine and Ca^2+^, respectively, were analyzed at optimized conditions to construct two calibration curves of two analytes (Figure 5). The regression equations of the calibration curves represent relationships between analyte concentrations (mg/L) and peak areas (mV.s). Good linearity was observed for both glucosamine (*R*^2^ = 0.9994), and Ca^2+^ (*R*^2^ = 0.9994) over the concentration ranges (Figure 5). Concentrations of analytes in analytical solutions (including real samples and standard addition samples) should be within the linear ranges.

#### 3.2.3. Limits of Detection and Quantification

Limits of detection (LODs) of glucosamine and Ca^2+^ were determined using signal-to-noise ratios equal to 3 (*S*/*N* = 3). A standard solution of each analyte was repeatedly diluted until its signals were 3 times higher than the background noise measured near the peak area (*S*/*N* = 3) (Table S9). The concentration of the analyte at *S*/*N* = 3 was recorded as the LOD value. LODs of glucosamine and Ca^2+^ were 1.00 and 0.05 mg/L, respectively. For the LOQ calculation, we did a similar experiment until the analyte's signal was 10 times higher than the background noise (Table S9). LOQs were estimated to be 3.30 mg/L for glucosamine and 0.17 mg/L for Ca^2+^.

The LOD of glucosamine obtained by our method was higher than that reported by Akamatsu and Mitsuhashi (0.01 mg/L) [51]. However, their method required in-capillary derivatization with *o*-phthalaldehyde, which may not be suitable for determining both Ca^2+^ and glucosamine in that condition. Our LOD value was comparable to the LODs of other studies using CE and HPLC (0.25 to 1.0 mg/L) [52, 53]. Our method not only detects both analytes in a single run but also achieves low LOD and LOQ, which are compatible with pharmaceutical and nutraceutical analysis.

#### 3.2.4. Repeatability

Standard solutions with three concentration levels of glucosamine and Ca^2+^ (20, 40, and 80 mg/L) were chosen for replication analysis (*n* = 5) for repeatability evaluation (Table S4). For Ca^2+^, relative standard deviations (RSD) of peak areas ranged from 0.92% to 2.16%. Otherwise, the RSD of the glucosamine peak areas ranged from 0.80% to 2.52%. Both glucosamine and Ca^2+^ migration times in standards had an RSD <2.5% at all concentration levels. These method repeatability results meet the standard precision criteria proposed by AOAC International for concentration ranges from 1 ppm (11%) to 100 ppm (5.3%) [54].

#### 3.2.5. Recovery

Recovery is an essential parameter in evaluating the method of accuracy. In this study, we used a tablet sample without glucosamine and Ca^2+^ (with no label claim of these components and confirmed with reference methods) to make matrix-spike samples. Three spiking levels of glucosamine (40, 80, and 100 mg/L), and Ca^2+^ (4, 8, and 10 mg/L) were performed in triplicate. The sample matrix (about 0.5 g) was spiked with a standard solution containing glucosamine and Ca^2+^, equilibrated for at least 30 min at room temperature, and treated according to the procedure described in Section 2.2. Recoveries of glucosamine and Ca^2+^ ranged from 96.9% to 102.0% and from 98.0% to 103.0%, respectively (see Table S5). These recovery rates satisfy the AOAC requirement for expected recovery at concentration levels from 10 ppm (80–100%) to 100 ppm (90–107%) [54], indicating high accuracy with an insignificant compound loss of our analytical procedure.

### 3.3. Method Application and Cross-Checked Results

**Figure 6:**
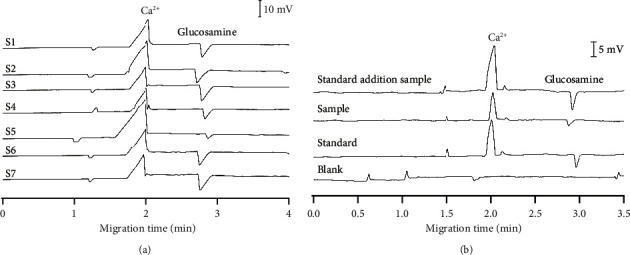
Representative CE-C^4^D electropherograms of (a) different supplement samples (Table 1). (b) Specific peaks evaluated by blank, standard, sample, and standard addition sample. BGE conditions: 10 mM Tris/Ace electrolyte solution, pH of 5.0, separation voltage of +20 kV, sample injection time of 30 s, sample injection height of 25 cm.

The validated method was applied to seven real samples collected from pharmacies in Hanoi, Vietnam, including four tablets and three hard capsules (Table 1 and Figure 6(a)). To verify if signal peaks from samples were generated by glucosamine and Ca^2+^, known amounts of standard solutions were added to the sample before analyzing by CE-C^4^D (Figure 6(b)). The intensities of both signals in the standard addition samples increased compared to those in the samples. The migration times of glucosamine and Ca^2+^ peaks were similar between standard and standard addition samples. It is shown that the supplement additives have a minimal impact on the migration time of both analytes. Thus, the content of glucosamine and Ca^2+^ in supplements could be determined using the standard addition method. Based on the peak area obtained and the standard addition equation, the content of calcium and glucosamine in the functional food samples will be calculated.

The contents of Ca^2+^ in the samples measured by our CE-C^4^D method ranged from 7.82 to 55.43 mg per unit (e.g., tablet or capsule), with relative deviations compared to label claims ranging from −7.62% to +5.20%. The contents of glucosamine ranged from 111.0 to 643.0 mg per unit, and measured-label deviations ranged from −5.26% to +8.10% (Table 1). There was an acceptable agreement between measured and label claim contents because deviations are less than ±10% in all samples. The cross-checked analysis was performed for all the samples (Table 1). The relative deviations between the results of CE and ICP-OES ranged from −8.08% to +5.62% for Ca^2+^, and between CE and HPLC, ranged from −5.93% to +6.47% for glucosamine. Good agreement between analyte contents measured by our method and the reference methods indicates that CE-C^4^D could be an alternative tool for conventional methods such as HPLC and ICP-OES in specific applications.

## 4. Conclusions

The present study developed an analytical method for the simultaneous determination of glucosamine and Ca^2+^ using CE-C^4^D in commercial dietary supplements. Our method was demonstrated to be simple, fast, and cost-effective with adequate validation parameters such as selectivity, linearity, detection limits, repeatability, and recovery. Concentrations of glucosamine and Ca^2+^ in supplement samples can be simultaneously determined in a single run analysis within five min. The validated method was applied to analyze samples, including tablets and hard capsules. Analytical results obtained by this CE-C^4^D method were in good agreement with labeling contents and those measured by reference methods (i.e., HPLC-FLD for glucosamine and ICP-OES for Ca^2+^). Overall, our study has revealed another application of CE-C^4^D as an alternative green analytical tool for conventional dietary supplement quality control methods, with the advantages of high efficiencies, low sample and electrolyte consumption, and minimal chemical waste. Because one simple analytical procedure can be applied for both analytes (organic compound and metal ion) with different properties, our method significantly reduced analysis time and labor cost compared to using one conventional method for each analyte. The proposed CE-C^4^D method for glucosamine and Ca^2+^ analysis also provided reliable results compared to standard techniques.

## Figures and Tables

**Table 1:** Determination of glucosamine and Ca^2+^ in commercial dietary supplements from pharmacies in Vietnam, using CE-C^4^D and HPLC, ICP-OES as the reference methods.

Sample no	Types of pills	Glucosamine					Ca^2+^				
		CE-C^4^D (mg/*μ*)	Label (mg/*μ*)	HPLC (mg/*μ*)	CE-LBL (%)	CE-HPLC (%)	CE-C^4^D (mg/*μ*)	Label (mg/*μ*)	ICP-OES (mg/*μ*)	CE-LBL (%)	CE-ICP (%)
*S*1	Tablet	479.0	488.0	450.0	−1.84	6.44	26.30	25.00	24.90	5.20	5.62
*S*2	Tablet	494.0	457.0	464.0	8.10	6.47	41.90	40.00	44.80	4.75	−6.47
*S*3	Tablet	616.0	625.0	613.0	−1.44	0.49	36.60	35.00	36.90	4.57	−0.81
*S*4	Tablet	223.0	211.0	230.0	5.69	−3.04	48.50	50.00	47.50	−3.00	2.11
*S*5	Hard capsule	111.0	107.0	118.0	3.74	−5.93	49.40	48.00	47.70	2.92	3.56
*S*6	Hard capsule	324.0	342.0	344.0	−5.26	−5.81	7.82	8.00	7.80	−2.25	0.26
*S*7	Hard capsule	643.0	616.0	676.0	4.38	−4.88	55.43	60.00	60.30	−7.62	−8.08

CE-LBL: CE-label deviation the deviation of the result obtained with CE-C^4^D from that indicated on the label; CE-LBL (%) = {(C_CE-C4D_ − C_label_)/C_label_} × 100%. CE-HPLC: CE-HPLC deviation, CE-ICP : CE-ICP deviation; they are the deviation of the result obtained with CE-C^4^D from that with the standard reference method (HPLC and ICP-OES); CE-HPLC (or CE-ICP) (%) = {(C_CE-C4D_ − C_reference_)/C_reference_} ‐ 100% mg/*μ* : mg per unit (e.g., tablet or capsule).

## Data Availability

The findings of the study are available from the corresponding author upon request.

## Supplementary Materials

Supplementary Materials Table S1. The background noise, peak area, and migration time of glucosamine and Ca^2+^ at different BGE conditions. Table S2: The background noise, peak area, and migration time of glucosamine and Ca^2+^ at different pH conditions. Table S3: The background noise, peak area, and migration time of glucosamine and Ca^2+^ at different Tris concentrations. Table S4: The background noise, peak area, and migration time of glucosamine and Ca^2+^ at different seperation voltages. Table S5: The background noise, peak area, and migration time of glucosamine and Ca^2+^ at different injection time (siphoning at 25 cm). Table S6: The background noise, peak area, and migration time of glucosamine and Ca^2+^ at different siphoning height (injection time 30 s). Table S7. The optimal conditions of the CE-C^4^D method for simultaneous determination of glucosamine and Ca^2+^. Table S8. The optimal conditions for determination of glucosamine by HPLC-FLD and Ca^2+^ by ICP-OES. Table S9. LOD and LOQ of glucosamine and Ca^2+^. Table S10. The repeatability evaluation of peak area (mV.s) and migration time (min) for simultaneous determination of glucosamine and Ca^2+^ by CE-C^4^D. Table S11. Recoveries for simultaneous determination of glucosamine and Ca^2+^ by CE-C^4^D.
