# Supplementary material for: Green Analytical Method for Simultaneous Determination of Glucosamine and Calcium in Dietary Supplements by Capillary Electrophoresis with Capacitively Coupled Contactless Conductivity Detection
Source: J Anal Methods Chem. 2023 Jan 31;2023:2765508. doi: 10.1155/2023/2765508 (PMC9904918; doi:10.1155/2023/2765508)
Supplement: Supplementary Materials — Table S1. The background noise, peak area, and migration time of glucosamine and Ca2+ at different BGE conditions. Table S2: The background noise, peak area, and migration time of glucosamine and Ca2+ at different pH conditions. Table S3: The background noise, peak area, and migration time of glucosamine and Ca2+ at different Tris concentrations. Table S4: The background noise, peak area, and migration time of glucosamine and Ca2+ at different seperation voltages. Table S5: The background noise, peak area, and migration time of glucosamine and Ca2+ at different injection time (siphoning at 25 cm). Table S6: The background noise, peak area, and migration time of glucosamine and Ca2+ at different siphoning height (injection time 30 s). Table S7. The optimal conditions of the CE-C4D method for simultaneous determination of glucosamine and Ca2+. Table S8. The optimal conditions for determination of glucosamine by HPLC-FLD and Ca2+ by ICP-OES. Table S9. LOD and LOQ of glucosamine and Ca2+. Table S10. The repeatability evaluation of peak area (mV.s) and migration time (min) for simultaneous determination of glucosamine and Ca2+ by CE-C4D. Table S11. Recoveries for simultaneous determination of glucosamine and Ca2+ by CE-C4D. [file 2765508.f1.docx]

**Supporting Information**

**Green analytical method for simultaneous determination of glucosamine and calcium in dietary supplements by capillary electrophoresis coupled with contactless conductivity detection**

Yen Nhi Do ^a^, Thi Lan Phuong Kieu ^a,b^, Thi Huyen My Dang ^a^, Quang Huy Nguyen ^a,c^, Thu Hien Dang ^b^**,** Cao Son Tran ^b^, Anh Phuong Vu ^d^, Thi Trang Do ^d^, Thi Ngan Nguyen ^d^, Son Luong Dinh ^d^, Thi Minh Thu Nguyen ^a^, Thi Ngoc Mai Pham ^a^, Anh Quoc Hoang ^a^, Bach Pham ^a,*^, Thi Anh Huong Nguyen ^a,^*

^a^ *Faculty of Chemistry, University of Science, Vietnam National University, Hanoi, 19 Le Thanh Tong, Hanoi 10000, Vietnam*

^b^ *National Institute for Food Control (NIFC), 65 Pham Than Duat, Hanoi 10000, Vietnam*

^c^ *Faculty of Pharmacy, University of Medicine and Pharmacy, Thai Nguyen University, 284 Luong Ngoc Quyen, Thai Nguyen 24000, Vietnam*

^d^ *Poison Control Center, Bach Mai Hospital, 78 Giai Phong, Hanoi 10000, Vietnam*

**Correspondence should be addressed to:**

Thi Anh Huong Nguyen ([nguyenthianhhuong@hus.edu.vn](mailto:nguyenthianhhuong@hus.edu.vn))

Bach Pham ([phamgiabach@hus.edu.vn](mailto:phamgiabach@hus.edu.vn))

**Table S1.** The background noise, peak area, and migration time of glucosamine and Ca^2+^ at different BGE conditions

| Analytes | Buffer | | | | | | | | | | | |
| --- | --- | --- | --- | --- | --- | --- | --- | --- | --- | --- | --- | --- |
|  | Tris/Ace | | | Arg/Ace | | | His/Ace | | | CAPS/Ace | | |
|  | Noise  (mV) | S_peak_ (mV.s) | Time  (min) | Noise  (mV) | S_peak_ (mV.s) | Time  (min) | Noise  (mV) | S_peak_ (mV.s) | Time  (min) | Noise  (mV) | S_peak_ (mV.s) | Time  (min) |
| Ca | 0.76 | 27.59 | 1.99 | 1.12 | 36.08 | 1.95 | 0.62 | 43.01 | 1.94 | 0.45 | 4.02 | 1.99 |
| Glucosamine |  | 13.47 | 2.60 |  | 2.47 | 2.71 |  | 1.51 | 2.71 |  | 2.35 | 2.67 |

| Analytes | pH | | | | | | | | | | | | | | |
| --- | --- | --- | --- | --- | --- | --- | --- | --- | --- | --- | --- | --- | --- | --- | --- |
|  | pH=4.5 | | | pH=5.0 | | | pH=5.5 | | | pH=6 | | | pH=6.5 | | |
|  | Noise  (mV) | S_peak_ (mV.s) | Time  (min) | Noise  (mV) | S_peak_ (mV.s) | Time  (min) | Noise  (mV) | S_peak_ (mV.s) | Time  (min) | Noise  (mV) | S_peak_ (mV.s) | Time  (min) | Noise  (mV) | S_peak_ (mV.s) | Time  (min) |
| Ca | 0.73 | 32.63 | 1.95 | 0.80 | 37.56 | 1.95 | 0.68 | 32.84 | 1.91 | 0.75 | 38.12 | 1.91 | 0.78 | 37.45 | 1.92 |
| Glucosamine |  | 12.66 | 2.78 |  | 14.57 | 2.60 |  | 16.05 | 2.62 |  | 11.96 | 2.50 |  | 12.57 | 2.52 |

**Table S2 :** The background noise, peak area, and migration time of glucosamine and Ca^2+^ at different pH conditions.

**Table S3:** The background noise, peak area, and migration time of glucosamine and Ca^2+^ at different Tris concentrations

| Analytes | Buffer concentration | | | | | | | | | | | |
| --- | --- | --- | --- | --- | --- | --- | --- | --- | --- | --- | --- | --- |
|  | 8 mM | | | 10 mM | | | 15 mM | | | 20 mM | | |
|  | Noise  (mV) | S_peak_ (mV.s) | Time  (min) | Noise  (mV) | S_peak_ (mV.s) | Time  (min) | Noise  (mV) | S_peak_ (mV.s) | Time  (min) | Noise  (mV) | S_peak_ (mV.s) | Time  (min) |
| Ca | 1.02 | 45.17 | 1.95 | 1.02 | 38.03 | 1.99 | 0.86 | 10.23 | 1.95 | 0.42 | 7.98 | 1.95 |
| Glucosamine |  | 18.78 | 2.70 |  | 14.45 | 2.59 |  | 5.01 | 2.59 |  | 3.74 | 2.62 |

**Table S4:** The background noise, peak area, and migration time of glucosamine and Ca^2+^ at different seperation voltages

| Analytes | Voltage | | | | | | | | | | | |
| --- | --- | --- | --- | --- | --- | --- | --- | --- | --- | --- | --- | --- |
|  | +10kV | | | +15 kV | | | +20 kV | | | +25 kV | | |
|  | Noise  (mV) | S_peak_ (mV.s) | Time  (min) | Noise  (mV) | S_peak_ (mV.s) | Time  (min) | Noise  (mV) | S_peak_ (mV.s) | Time  (min) | Noise  (mV) | S_peak_ (mV.s) | Time  (min) |
| Ca | 0.67 | 50.55 | 2.21 | 1.21 | 37.51 | 1.85 | 1.53 | 32.02 | 1.41 | 2.06 | 26.03 | 1.23 |
| Glucosamine |  | 23.02 | 4.49 |  | 16.03 | 3.31 |  | 15.07 | 2.97 |  | 10.03 | 2.15 |

**Table S5:** The background noise, peak area, and migration time of glucosamine and Ca^2+^ at different injection time (siphoning at 25 cm)

| Analytes | Injection time | | | | | | | | | | | |
| --- | --- | --- | --- | --- | --- | --- | --- | --- | --- | --- | --- | --- |
|  | 20 s | | | 25 s | | | 30 s | | | 35 s | | |
|  | Noise  (mV) | S_peak_ (mV.s) | Time  (min) | Noise  (mV) | S_peak_ (mV.s) | Time  (min) | Noise  (mV) | S_peak_ (mV.s) | Time  (min) | Noise  (mV) | S_peak_ (mV.s) | Time  (min) |
| Ca | 1.05 | 12.20 | 1.78 | 0.85 | 25.04 | 1.82 | 0.83 | 33.12 | 1.88 | 0.80 | 48.24 | 1.90 |
| Glucosamine |  | 5.32 | 2.75 |  | 10.34 | 2.96 |  | 15.65 | 2.99 |  | 20.05 | 2.99 |

**Table S6:** The background noise, peak area, and migration time of glucosamine and Ca^2+^ at different siphoning height (injection time 30 s)

| Analytes | Siphoning height | | | | | | | | |
| --- | --- | --- | --- | --- | --- | --- | --- | --- | --- |
|  | 20 cm | | | 25 cm | | | 30 cm | | |
|  | Noise  (mV) | S_peak_ (mV.s) | Time  (min) | Noise  (mV) | S_peak_ (mV.s) | Time  (min) | Noise  (mV) | S_peak_ (mV.s) | Time  (min) |
| Ca | 0.80 | 25.15 | 1.89 | 0.83 | 33.12 | 1.88 | 0.87 | 47.89 | 1.88 |
| Glucosamine |  | 10.23 | 2.99 |  | 15.65 | 2.99 |  | 20.14 | 2.99 |

**Table S7.** The optimal conditions of CE-C^4^D method for simultaneous determination of glucosamine and Ca^2+^

| **Factors** | **Optimal conditions** |
| --- | --- |
| Detector | CE-C^4^D |
| Capillary | Silica capillary, total length of 40cm, effective length of 30cm, inner diameter of 50µm |
| Method of injection | Hydrodynamic (siphoning) |
| Electrolyte solution | Tris/Ace 10mM, pH of 5.0 |
| Separation voltage | +20kV |
| Sample injection time | 30s, siphoning at 25 cm height |

**Table S8.** The optimal conditions for determination of glucosamine by HPLC-FLD and Ca^2+^ by ICP-OES

| **Reference methods for determination of glucosamine (HPLC-FLD)** | **Reference methods for determination of Ca^2+^**  **(ICP-OES)** |
| --- | --- |
| Detection mode: Fluorescence  Excitation wavelength λ_ex_ 265 nm  Emission wavelength λ_em_ 315 nm  C18 column (150 mm x 4.6 mm x 5 µm) (Waters Corporation)  Flow rate: 1 mL/min  Injection volume: 20 µL  HPLC Gradient Program   \| Time (min) \| A (H_2_O) (%) \| B (Acetonitrile) (%) \| \| --- \| --- \| --- \| \| 0.01 \| 70 \| 30 \| \| 6.00 \| 10 \| 90 \| \| 8.00 \| 10 \| 90 \| \| 8.01 \| 70 \| 30 \| \| 13.00 \| 70 \| 30 \|   Pre-column derivatization of glucosamine with 9-fluorenylmethoxycarbonyl chloride (FMOC) :  - 200 µL of the sample was first accurately pipetted to a 1.8 mL vial, then 400 µL borate buffer (pH = 8.0) and 400 µ of FMOC 500 ppm were added. The mixture was shaken for around 10 s before being allowed to react at room temperature for at least 30 min. Finally, the reaction mixture was analyzed by HPLC-FLD | Power: 1.1 kW  Plasma gas flow: 15.0 L/min  Auxiliary gas flow: 0.2 L/min  Wavelength: 317.933 nm  Nebulizer: 0.8 L/min  Plasma view: Radial  Sample flow rate: 1.5 mL/min  Delay time: 30 s |

**Table S9.** LOD and LOQ of glucosamine and Ca^2+^

| **Analytes** | **Conc. (mg/L)** | **Peak height (S)**  **(mV)** | **Background noise signal (N)**  **(mV)** | **S/N** | **LOD (mg/L)** | **LOQ (mg/L)** |
| --- | --- | --- | --- | --- | --- | --- |
| Glucosamine | 1.00 | 0.52 | 0.17 | 3.06 | **1.00** | **3.30** |
| Calcium | 0.05 | 0.63 | 0.20 | 3.15 | **0.05** | **0.17** |

**Table S10.** The repeatability evaluation of peak area (mV.s) and migration time (min) for simultaneously determination of glucosamine and Ca^2+^ by CE-C^4^D

| **Analyte** | **Conc. (mg/L)** | **Peak area (mV.s)** | | | | | **Average peak area (mV.s)** | **RSD (%)** | **Migration time (min)** | | | | | **Average**  **(min)** | **RSD (%)** |
| --- | --- | --- | --- | --- | --- | --- | --- | --- | --- | --- | --- | --- | --- | --- | --- |
|  |  | **1** | **2** | **3** | **4** | **5** |  |  | **1** | **2** | **3** | **4** | **5** |  |  |
| Ca^2+^ | 20.00 | 39.20 | 38.20 | 40.10 | 40.50 | 39.50 | 40.30 | **2.16** | 1.87 | 1.84 | 1.85 | 1.82 | 1.80 | 1.84 | **1.13** |
|  | 40.00 | 59.70 | 58.90 | 60.70 | 58.80 | 59.70 | 59.60 | **1.15** | 1.86 | 1.89 | 1.87 | 1.88 | 1.88 | 1.88 | **0.47** |
|  | 80.00 | 136.50 | 134.50 | 138.40 | 136.10 | 136.70 | 136.40 | **0.92** | 1.95 | 1.90 | 1.86 | 1.89 | 1.91 | 1.90 | **1.18** |
| Glucosamine | 20.00 | 2.17 | 2.06 | 2.15 | 2.19 | 2.09 | 2.08 | **2.52** | 2.89 | 2.90 | 2.92 | 2.95 | 2.92 | 2.92 | **0.58** |
|  | 40.00 | 4.74 | 4.85 | 4.73 | 4.87 | 4.88 | 4.79 | **1.37** | 2.89 | 2.97 | 2.93 | 2.98 | 3.01 | 2.96 | **1.24** |
|  | 80.00 | 9.13 | 9.26 | 9.22 | 9.10 | 9.09 | 9.23 | **0.80** | 2.92 | 2.89 | 3.12 | 3.00 | 2.98 | 2.98 | **2.09** |

**Table S11.** Recoveries for simultaneously determination of glucosamine and Ca^2+^ by CE-C^4^D

| **Analyte** | **No.** | **Standard concentration added (mg/L)** | **Recovery concentration (mg/L)** | **Recovery (%)** | **Average recovery (%)** |
| --- | --- | --- | --- | --- | --- |
| Ca^2+^ | 1 | 4.00 | 3.92 | 98.0 | **99.8** |
|  | 2 | 8.00 | 8.22 | 103 |  |
|  | 3 | 10.0 | 9.85 | 98.5 |  |
| Glucosamine | 1 | 40.0 | 40.8 | 102 | **100.0** |
|  | 2 | 80.0 | 77.5 | 96.9 |  |
|  | 3 | 100 | 101 | 102 |  |
